# Creation and validation of a mortality risk prediction model for ICU patients with traumatic brain injury: a multicenter retrospective cohort study

**DOI:** 10.1186/s40001-025-03656-3

**Published:** 2025-12-09

**Authors:** Wenchao Wu, Qingsong Li

**Affiliations:** Department of Neurosurgery, The Second Affiliated Hospital of Harbin Medical University, Harbin Medical University, No. 157, Health Care Road, Harbin, Heilongjiang China

**Keywords:** Traumatic brain injury, Nomogram, External validation, Short-term mortality prediction, Mortality

## Abstract

**Background:**

Traumatic brain injury (TBI) stands as a major global cause of mortality and disability. Accurate prediction of in-hospital mortality is crucial for optimizing clinical management of TBI patients in the intensive care unit (ICU). However, existing prognostic models demonstrate significant limitations in short-term prediction and clinical immediacy. This study aims to develop and validate a practical prognostic model to address these research gaps and provide clinicians with a precise risk stratification tool.

**Methods:**

This study integrated data from two publicly available databases, MIMIC-IV and eICU–CRD. The MIMIC-IV database was utilized for model development and internal validation, while the eICU–CRD database was employed for external validation. The research enrolled TBI patients admitted to the intensive care unit as study subjects. During the data preprocessing phase, continuous variables with missing rates below 30% were handled using multiple imputation, while missing values in categorical variables were retained as a separate category. Variables with missing rates exceeding 30% were excluded. Subsequently, eligible cases from the MIMIC-IV database were randomly divided into training and testing sets at a 7:3 ratio. Based on multiple regression analysis supplemented by LASSO regression screening, we developed a risk assessment model to identify independent predictors of short-term mortality in ICU-admitted TBI patients. Finally, the model's performance was systematically evaluated across three dimensions: discrimination, calibration, and clinical utility.

**Results:**

Strictly adhering to the inclusion and exclusion criteria, we ultimately enrolled 3604 TBI patients across the two databases. The final model incorporated seven independent predictors: APS-III score, age, use of mechanical ventilation, respiratory rate, prothrombin time, sodium level, and anion gap. In the training set, the 7-day mortality prediction model demonstrated excellent discriminative ability, with an AUC value of 0.829 (95% CI 0.803–0.855), a sensitivity of 78.6%, and a specificity of 72.6%. The model's performance further improved in the test set, achieving an AUC value of 0.871 (95% CI 0.822–0.921), with sensitivity and specificity increasing to 83.1% and 77.6%, respectively. During external validation, the model also exhibited robust predictive performance, yielding an AUC value of 0.757 (95% CI 0.711–0.803) for 7-day mortality, along with a sensitivity of 67.3% and a specificity of 74.8%, further confirming its generalizability. Following bootstrap internal validation, the predictive model demonstrated excellent performance. It exhibited strong discriminatory power (corrected AUC = 0.8309, 95% CI 0.8241–0.8356) and favorable overall predictive accuracy (Brier score = 0.1126, 95% CI 0.1117–0.1138). Calibration analysis confirmed model reliability: the calibration intercept approached zero (0.0068, 95% CI 0.0078–0.0219), indicating no systematic overestimation or underestimation, while the calibration slope approached unity (0.9622, 95% CI 0.8677–1.0668), demonstrating excellent alignment between predicted probability ranges and actual risk variations. These metrics collectively demonstrate the model's strong clinical applicability, and its predictions can be reliably used to guide clinical decision-making. The calibration curve also demonstrated high consistency, while decision analysis revealed significant clinical net benefit across different risk thresholds.

**Conclusions:**

This study developed a predictive model that estimates short-term mortality for TBI patients in the ICU using seven routinely available clinical variables. The model demonstrated robust performance in external validation. Its design, which enables multi-timepoint assessment, may facilitate risk stratification and has the potential to support clinical decision-making, pending future prospective validation.

**Supplementary Information:**

The online version contains supplementary material available at 10.1186/s40001-025-03656-3.

## Introduction

TBI is defined as a major public health challenge. Its pathological basis involves structural and functional impairments of the brain caused by external mechanical forces acting on the head. Such damage may be temporary or permanent and often results in high rates of disability [1]. TBI is one of the leading causes of death and disability worldwide. In the United States alone, 2014 surveillance data illustrated the substantial burden of TBI, accounting for approximately 2.8 million emergency department visits, 288,000 hospitalizations, and 56,800 deaths across all age groups [2–4]. TBI epidemiology shows distinct patterns, with the highest emergency department visit rates among adults aged 75 and older, followed by children aged 0–4 and adolescents/young adults aged 15–24 [1, 4].

Patients who sustain severe TBI often require ICU admission for advanced monitoring and organ support. These individuals exhibit diverse clinical symptoms and face a significant risk of adverse outcomes, highlighting the necessity for early identification of high-risk cases. Although prevention strategies and clinical management for TBI have seen progressive refinement, significant challenges remain in improving final patient outcomes, and current treatment efficacy is often suboptimal [1]. The pathophysiology of TBI is highly complex and dynamic, involving primary and secondary injury mechanisms influenced by numerous risk factors. Traditional indicators such as vital signs, presence of brain herniation, relevant scoring systems, and imaging features are all recognized prognostic factors. Recent studies, however, have focused on exploring the prognostic significance of various biomarkers and laboratory parameters.

Despite the development of numerous prediction models designed to assess prognosis and mortality in TBI patients, only a minority of these tools are acknowledged by clinicians as having achieved their intended objectives or providing clinically valuable insights [5–7]. This gap highlights a significant unmet need for robust, accurate, and clinically acceptable predictive tools that can reliably stratify risk and inform decision-making for TBI patients. In recent years, the application of explainable machine learning in medical prognostic prediction has gained increasing attention, offering novel solutions to enhance the transparency of clinical decision-making [8, 9].

This study aims to identify predictors of mortality in TBI patients admitted to the ICU through multivariate regression analysis and to develop a corresponding prediction model. Using the eICU database for external validation, we sought to rigorously evaluate the model's generalizability. Ultimately, this study provides clinicians with a validated tool for mortality risk stratification, supporting clinical decision-making through objective risk assessment.

## Methods

### Data source

This study collected data from two publicly available intensive care unit databases: the eICU–CRD database and the MIMIC-IV database. The eICU–CRD (version 2.0) is a comprehensive database containing data from over 200,000 ICU admissions across 208 U.S. hospitals during 2014–2015 [10]. MIMIC-IV (v3.1) is a comprehensive single-center database comprising data from over 70,000 ICU stays at Beth Israel Deaconess Medical Center in Boston, MA, USA, spanning 2008–2019 [11]. Both databases offer deidentified clinical data encompassing patient demographics, vital signs, laboratory results, diagnostic codes (ICD-9 and ICD-10), treatment details, and clinical outcomes. The author has completed training and obtained database access (Record ID 67106156). The eICU–CRD has been certified by Privacert (Cambridge, MA) as meeting the de-identification standards specified in the HIPAA Privacy Rule Safe Harbor provisions (Certification No.: 1031219-2). The MIMIC-IV and eICU–CRD databases are derived from distinct healthcare systems and time periods, with no overlapping patient populations. This ensures the independence of the development and external validation cohorts, a crucial prerequisite for a rigorous assessment of the model's generalizability. This study employs a retrospective design and utilizes de-identified pre-existing data, thus requiring no additional Institutional Review Board review.

### Study patients

**Fig. 1:**
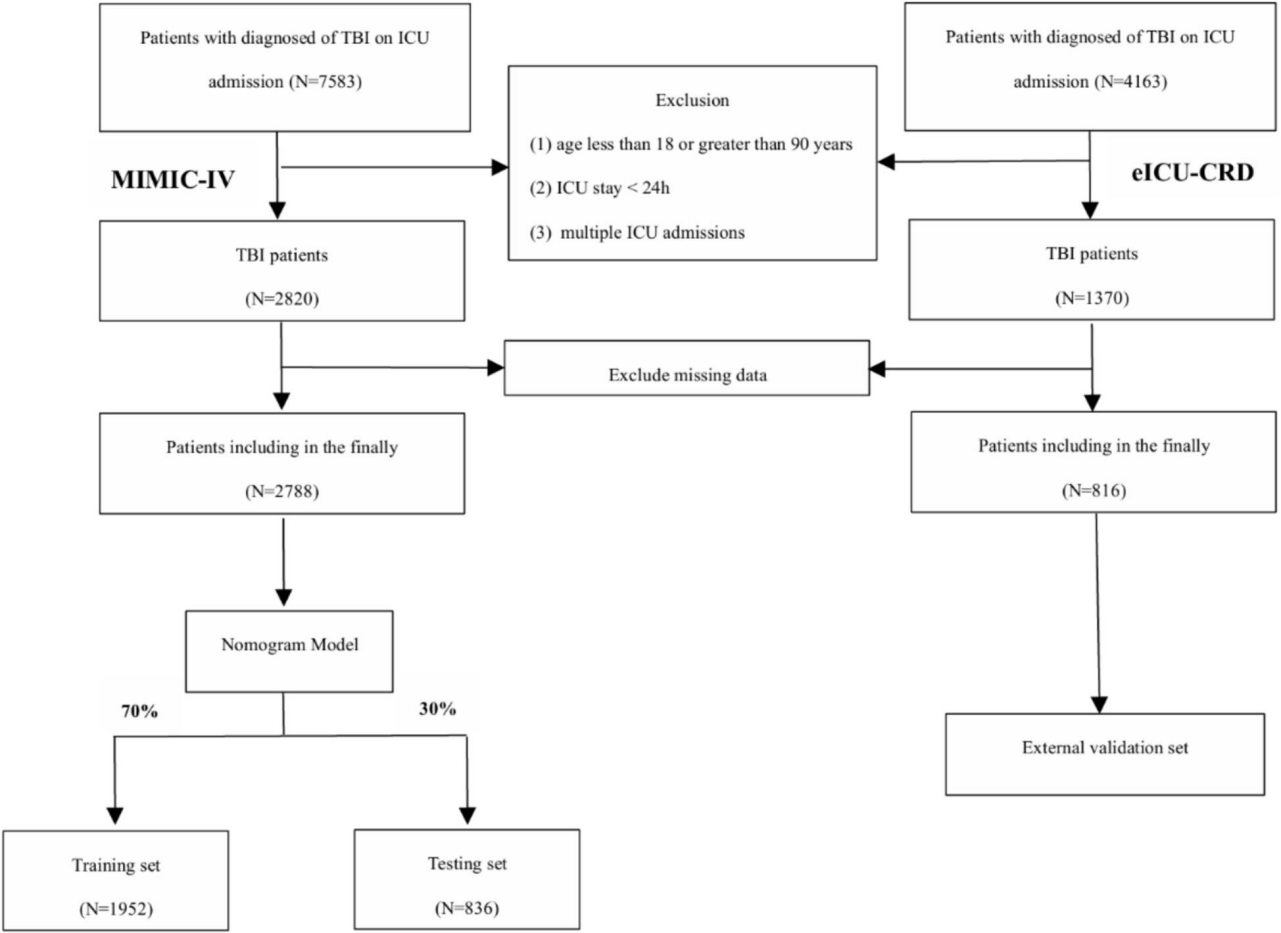
Flowchart of study population selection

TBI patients were identified through specific ICD-9 and ICD-10 codes in the database (ICD-9: 850.*–854.* and ICD-10: S06.*). Inclusion criteria: (1) initial admission to the intensive care unit (ICU); (2) Diagnosis of TBI within 48 h of admission; (3) Age ≥ 18 years. Patients were excluded based on the following criteria: (1) age under 18 or over 90 years; (2) an ICU stay of less than 24 h or multiple ICU admissions; and (3) absence of critical data required for model development and validation. The study population comprised 3,604 patients drawn from two databases. Subjects from the MIMIC-IV database were divided into a training set (70%) and an internal validation set (30%) for model development and validation. The independent cohort of 816 patients from the eICU–CRD served as an external validation set. The flowchart of study population selection is shown in Fig. 1.

### Data collection and variable extraction

Data were collected on general demographics, vital signs, laboratory parameters, treatments, comorbidities, and scoring systems. Variables were excluded if they had a missing value rate over 30% or were absent from both databases (Supplementary Figure S1) [12]. In addition, we adhered to the Events Per Variable (EPV) > 10 criterion throughout model development to ensure robustness, as is standard practice for predictive modeling with existing data sets, where a priori sample size calculation is not always feasible. Appropriate adjustments were made to the data collection process to ensure data quality and consistency. Following the stated principles, baseline variables within 24 h of ICU admission were extracted from the database, including gender, age, and ethnicity. Vital signs comprised heart rate (HR), respiratory rate (RR), temperature (T), oxygen saturation (SpO₂), and mean arterial pressure (MAP). Laboratory parameters encompass platelet count, white blood cell (WBC) count, red blood cell (RBC) count, bicarbonate, sodium (Na⁺), anion gap, potassium (K⁺), glucose, creatinine (Cr), and blood urea nitrogen (BUN). Comorbidities cover congestive heart failure, liver disease, renal disease, malignant cancer, and metastatic solid tumor. Interventions: mechanical ventilation. Disease severity was assessed using the Glasgow Coma Scale (GCS), Oxford Acute Severity of Illness Score (OASIS), and Simplified Acute Physiology Score III (SAPS III). Data extraction utilized structured query language (SQL) in PostgreSQL (v17.0) and Navicat Premium (v17.0.8).

### Research findings

The primary outcomes of this study were all-cause mortality at 7 days, 14 days, and 28 days following ICU admission. The selection of these timepoints was designed to align with key pathophysiological phases of mortality risk after TBI: 7-day mortality reflects early fatal brain injury, 14-day mortality assesses the impact of intermediate complications, and 28-day mortality serves as a standard endpoint in critical care research. Compared with in-hospital mortality, these landmark timepoints avoid biases introduced by inter-hospital variations in length of stay.

### Statistical analysis

Continuous variables were assessed for normality using the Kolmogorov–Smirnov test. Data meeting normality requirements were expressed as mean ± standard deviation and analyzed using Student's *t* test or analysis of variance (ANOVA). Non-normally distributed data were expressed as median (interquartile range) and analyzed using the Mann–Whitney *U* test or Kruskal–Wallis test. Categorical variables were expressed as frequencies (percentages), with intergroup comparisons performed using chi-square tests or Fisher's exact tests (as appropriate). Multicollinearity among variables was assessed using variance inflation factors (VIF).

To optimize the model structure and mitigate overfitting risks, we implemented a two-stage variable selection strategy. First, Least Absolute Shrinkage and Selection Operator (LASSO) regression was applied for preliminary screening. All continuous predictors were standardized, while categorical variables were incorporated into the model as dummy variables. The optimal penalty parameter was determined through tenfold cross-validation, ultimately selecting the lambda value within one standard error (*λ* = 0.023) to obtain a more concise variable set with enhanced generalizability. Subsequently, the variables selected by LASSO were incorporated into a multivariable logistic regression model to confirm their statistical significance as independent predictors [13]. To address potential class imbalance in outcome events, we employed standardized performance metrics including the standardized Brier score to ensure robust model evaluation. To evaluate the accuracy of the predictive model, we employed a ROC curve and quantified the model's predictive capability using the AUC value [14]. All analyses were performed using R software (version 4.5.1), with *p* < 0.05 (two-tailed) set as the threshold for statistical significance.

## Results

### Baseline characteristics from the MIMIC-IV

The study included 2788 patients from the MIMIC-IV database, selected according to predefined inclusion and exclusion criteria. The 28-day mortality rate was 16.03%, with 447 patients not surviving and 2341 surviving. The cohort had a mean age of 61.3 ± 21.3 years, with 63.7% (1775) being Female. Patients were randomly allocated to either the training set (1952 patients) or the testing set (836 patients) in a 7:3 ratio. Table 1 highlights significant differences in baseline characteristics between the two groups. Group of death were older and exhibited greater disease severity than survivors, as evidenced by significantly higher APS-III and OASIS scores, and lower GCS scores. Furthermore, the non-survivor group showed a higher prevalence of multiple organ dysfunction, including coagulation abnormalities, renal impairment, metabolic acidosis, and hemodynamic instability, along with a greater need for mechanical ventilation. The death group exhibited a higher prevalence of comorbidities, including heart disease, cerebrovascular disease, and malignant tumors. For a more detailed presentation of participant characteristics, Supplementary Table S1 provides outcome-stratified comparisons within the training cohort, and Supplementary Table S2 compares the distribution patterns of the training and testing data sets. No significant differences were observed in the baseline characteristics between the two data sets (*p* > 0.05), indicating that the two groups are sufficiently comparable.

**Table 1:** Baseline demographic and clinical characteristics of the study participants with TBI

Variable	Survival (*n* = 2341)	Non-survival (*n* = 447)	*p* value
Sex			
Male	825 (35.2%)	188 (42.1%)	0.007
Female	1516 (64.8%)	259 (57.9%)	
Race			
Caucasians	1456 (62.2%)	250 (55.9%)	0.015
Other	885 (37.8%)	197 (44.1%)	
Age (years)	59.53 ± 21.2	70.28 ± 19.66	< 0.001
PT(s)	13.35 ± 3.58	15.49 ± 6.98	< 0.001
PTT(s)	28.76 ± 7.23	31.75 ± 11.45	< 0.001
Glucose (mmol/L)	7.46 ± 3.03	8.69 ± 3.52	< 0.001
Na^+^ (mmol/L)	139.03 ± 4.11	140.35 ± 5.37	< 0.001
K^+^ (mmol/L)	4.09 ± 0.53	4.21 ± 0.62	< 0.001
HCO₃⁻ (mmol/L)	20.38 ± 6.13	23.61 ± 9.01	< 0.001
Cl^−^ (mmol/L)	104.01 ± 5.15	105.43 ± 6.48	< 0.001
RBC (× 10⁶/µL)	3.68 ± 0.72	3.46 ± 0.71	< 0.001
HR (minute)	82.02 ± 15.38	85.45 ± 16.59	< 0.001
SBP (mmHg)	124.68 ± 13.55	123.27 ± 14.36	0.056
DBP (mmHg)	66.41 ± 10.52	62.51 ± 10.54	< 0.001
RR (minute)	18.19 ± 2.99	19.68 ± 3.73	< 0.001
T (℃)	37.05 ± 0.45	37 ± 0.71	0.155
Spo2 (%)	97.44 ± 1.74	97.89 ± 2	< 0.001
APSIII score	35.68 ± 14.51	52.18 ± 22.14	< 0.001
OASIS score	30.44 ± 7.11	36.74 ± 7.4	< 0.001
GCS score	13.22 ± 2.4	12.12 ± 3.9	< 0.001
CCI score	3.21 ± 2.75	4.77 ± 2.97	< 0.001
Platelets (× 10^3^/µL)	206.37 ± 80.99	190.88 ± 84.68	< 0.001
WBC (× 10^3^/µL)	11.37 ± 5.83	13.13 ± 6.78	< 0.001
Anion gap (mEq/L)	14.32 ± 3.09	15.33 ± 3.46	< 0.001
Creatinine (mg/dL)	1.03 ± 0.9	1.28 ± 0.94	< 0.001
BUN (mg/dL)	17.39 ± 11.9	24.97 ± 17.98	< 0.001
Mechanical			
NO	1390 (59.4%)	151 (33.8%)	< 0.001
YES	951 (40.6%)	296 (66.2%)	
CHF			
NO	2086 (89.1%)	358 (80.1%)	< 0.001
YES	255 (10.9%)	89 (19.9%)	
CVD			
NO	2116 (90.4%)	380 (85%)	0.001
YES	225 (9.6%)	67 (15%)	
Chronic lung disease			
NO	2057 (87.9%)	388 (86.8%)	0.582
YES	284 (12.1%)	59 (13.2%)	
Malignant			
NO	2264 (96.7%)	419 (93.7%)	0.004
YES	77 (3.3%)	28 (6.3%)	
Liver			
NO	2203 (94.1%)	411 (91.9%)	0.105
YES	138 (5.9%)	36 (8.1%)	
MST			
NO	2321 (99.1%)	437 (97.8%)	0.02
YES	20 (0.9%)	10 (2.2%)	
AKI			
NO	798 (34.1%)	74 (16.6%)	< 0.001
YES	1543 (65.9%)	373 (83.4%)	

*MST* metastatic solid tumor, *AKI* acute kidney injury, *CVD* Cerebrovascular disease, *AG* anion gap

### Logistic regression analysis

To optimize the model structure and mitigate overfitting risk, this study employed LASSO regression for preliminary variable screening (Supplementary Figures S2, S3). With the optimal penalty parameter (*λ* = 0.023) determined via tenfold cross-validation, 12 clinical indicators were initially selected. Subsequently, multivariable Logistic regression was applied to further identify independent predictors for the model. The final predictive model incorporated the following seven core variables: age, mechanical ventilation, APS-III score, respiratory rate, anion gap, prothrombin time, and sodium level. Logistic regression analyses, both univariable and multivariable, were performed to determine factors linked to mortality in TBI patients. Table 2 identifies seven independent prognostic factors: age [OR 1.04, 95% CI 1.04–1.05, *p* < 0.001], mechanical ventilation [OR 3.30, 95% CI 2.38–4.57, *p* < 0.001], APS-III score [OR 1.03, 95% CI 1.02–1.04, *p* < 0.001], respiratory rate [OR 1.11, 95% CI 1.07–1.16, *p* = 0.045], anion gap [OR 1.07, 95% CI 1.03–1.12, *p* < 0.001], prothrombin time [OR 1.07, 95% CI 1.04–1.11, *p* < 0.001], and sodium level [OR 1.06, 95% CI 1.03–1.09, *p* < 0.001]. The VIF for all included variables was below 4 (APS-III: 1.101; age: 1.247; mechanical ventilation: 1.424; respiratory rate: 1.180; prothrombin time: 1.030; sodium: 1.042; anion gap: 1.027), indicating no significant multicollinearity concerns in the final regression model.

**Table 2:** Univariate and multivariate analyses of prognostic factors for 28-day mortality

Variable	Univariate analysis			Multivariate analysis		
	OR	95% CI	*p* value	OR	95% CI	*p* value
Sex	1.49	1.17–1.88	0.001	1.2	0.89–1.61	0.221
Race	0.75	0.59–0.95	0.016	0.8	0.60–1.07	0.125
Age	1.03	1.02–1.04	< 0.001	1.05	1.03–1.06	< 0.001
PT	1.11	1.08–1.15	< 0.001	1.06	1.03–1.10	< 0.001
PTT	1.03	1.02–1.05	< 0.001	1.01	0.99–1.02	0.325
Glucose	1.13	1.09–1.18	< 0.001	1.02	0.97–1.07	0.429
Na^+^	1.06	1.03–1.09	< 0.001	1.14	1.06–1.23	< 0.001
K^+^	1.44	1.17–1.76	< 0.001	1.31	1.02–1.68	0.032
RBC	0.66	0.56–0.78	< 0.001	1.21	0.98–1.50	0.071
HR	1.01	1.01–1.02	< 0.001	1.01	1.00–1.02	0.176
DBP	0.97	0.95–0.98	< 0.001	1.01	0.99–1.02	0.33
RR	1.15	1.11–1.19	< 0.001	1.12	1.06–1.17	< 0.001
Spo2	1.14	1.07–1.23	< 0.001	1.14	1.04–1.24	0.004
APSIII	1.05	1.04–1.05	< 0.001	1.04	1.02–1.05	< 0.001
OASIS	1.11	1.10–1.13	< 0.001	0.96	0.93–0.99	0.009
GCS	0.91	0.87–0.94	< 0.001	1.01	0.95–1.07	0.833
CCI	1.20	1.16–1.25	< 0.001	1.05	0.96–1.16	0.281
Platelets	1.00	1.00–1.00	< 0.001	1.00	1.00–1.00	0.146
WBC	1.04	1.02–1.06	< 0.001	1.02	1.00–1.04	0.126
AG	1.11	1.08–1.15	< 0.001	1.07	1.02–1.12	0.006
Creatinine	1.26	1.13–1.40	< 0.001	0.91	0.76–1.07	0.265
Mechanical	2.92	2.29–3.75	< 0.001	5.07	3.22–8.05	< 0.001
CHF	1.94	1.43–2.61	< 0.001	0.87	0.57–1.31	0.515
CVD	1.49	1.05–2.08	0.023	0.99	0.64–1.50	0.953
Malignant	2.00	1.18–3.28	0.008	1.06	0.54–2.04	0.854
Liver	1.66	1.07–2.51	0.02	1.05	0.57–1.86	0.88
MST	3.10	1.28–7.14	0.009	2.39	0.75–7.43	0.134
AKI	2.94	2.16–4.08	< 0.001	1.62	1.13–2.36	0.01

### Model construction and validation

**Fig. 2:**
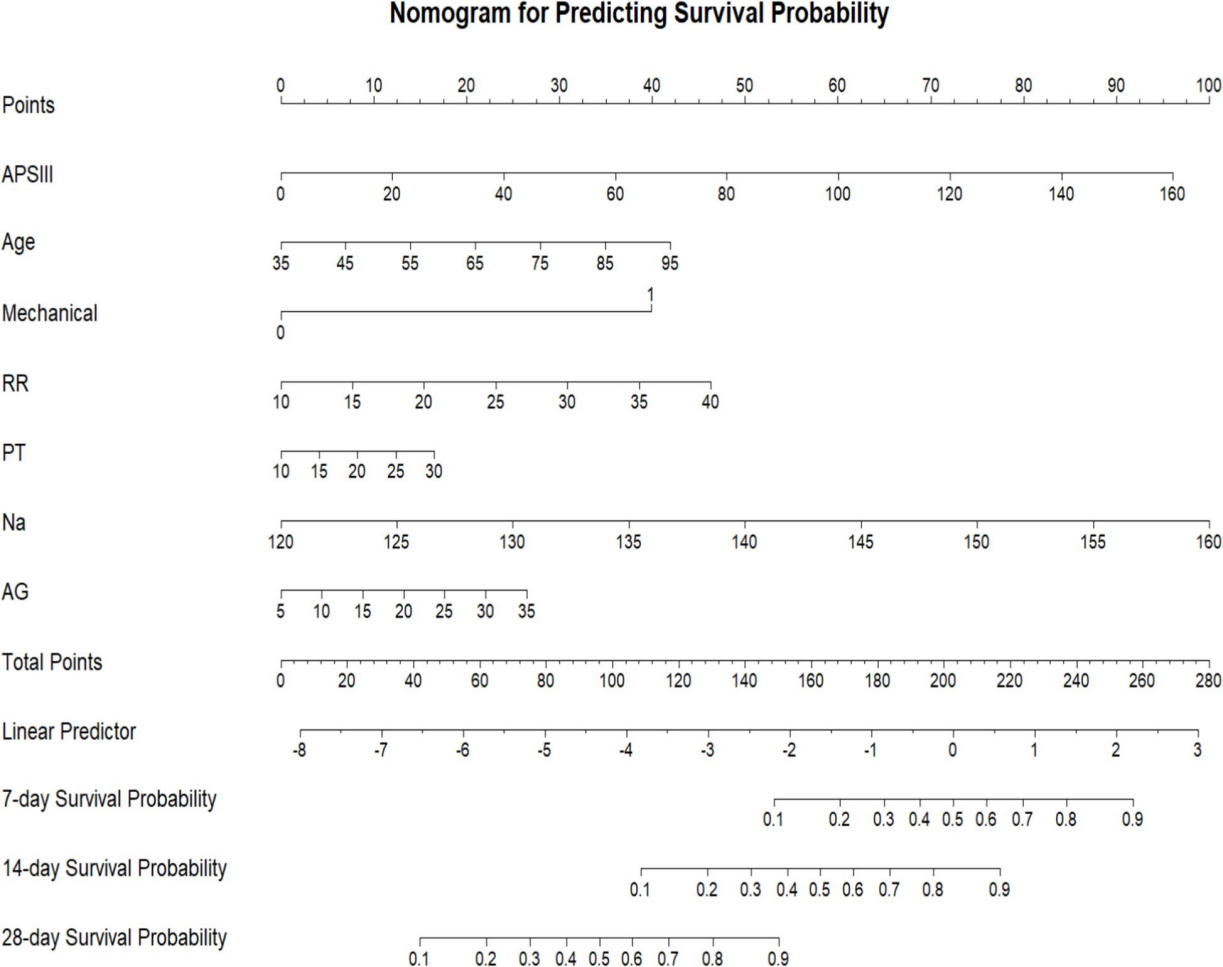
Short-term survival probability predicted by the nomogram at 7, 14, and 28 days after ICU admission

A nomogram was developed based on the seven independent predictors identified through regression analysis to estimate the probabilities of 7-day, 14-day, and 28-day survival in TBI patients (Fig. 2). The nomogram assigns specific points for each variable, and the total points correspond to predicted survival probabilities at each timepoint.

**Fig. 3:**
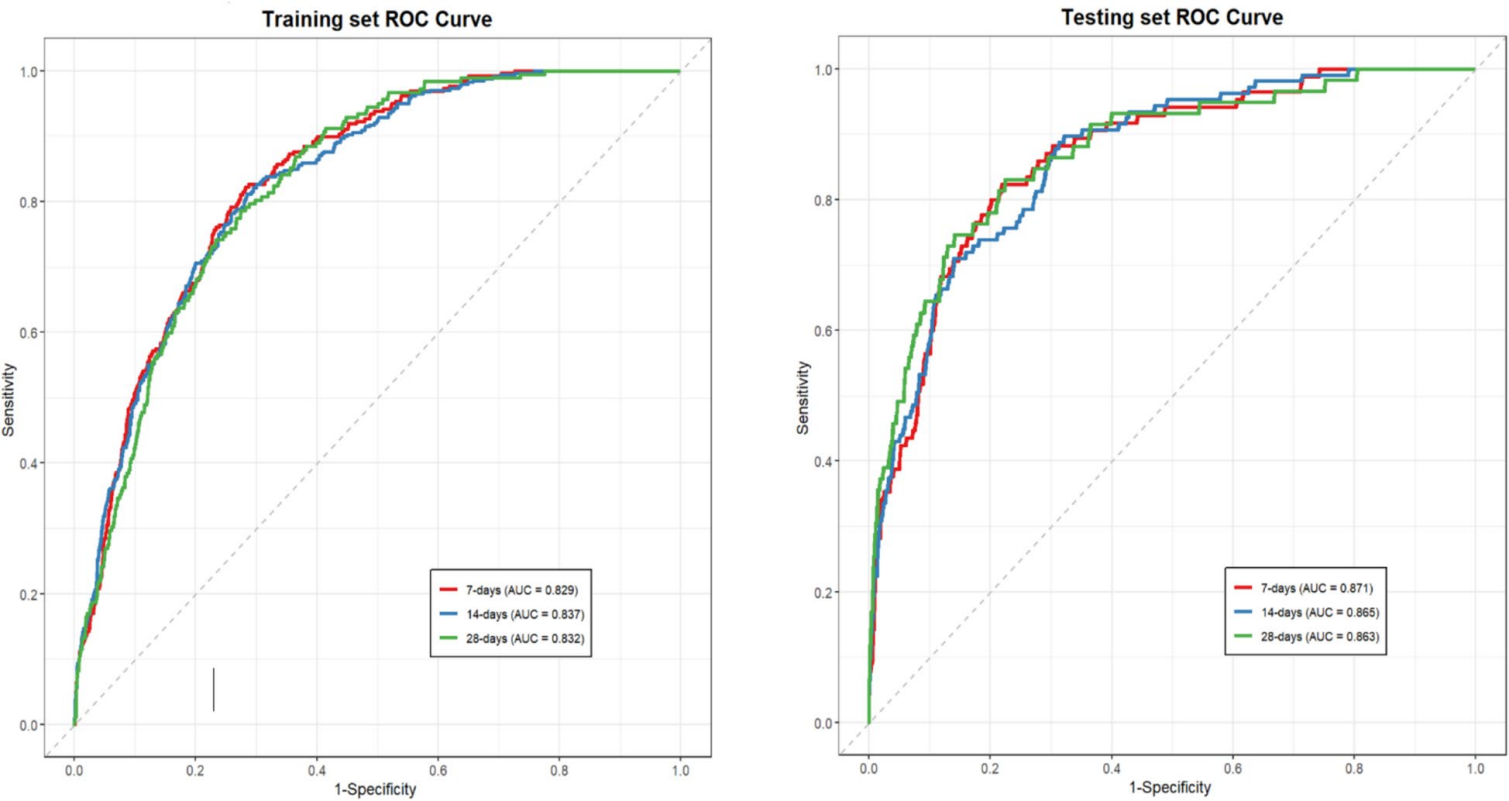
ROC curves of the nomogram model

The model's performance was evaluated through ROC curve, calibration curve, and decision curve analysis. Figure 3 demonstrates the predictive model's outstanding discriminatory performance in both the training and testing cohorts. Within the training set, the AUC values for predicting 7-day, 14-day, and 28-day mortality rates were 0.829, 0.837, and 0.832, respectively. In the testing set, these values improved to 0.871, 0.865, and 0.863. In the TBI cohort of this study, our proposed model (AUC = 0.832) demonstrated comparative performance in predicting 28-day mortality compared to two internationally established prognostic models—the IMPACT [15]core model (AUC = 0.631) and the CRASH basic model [16] (AUC = 0.587). These results suggest the potential value of our model, particularly its applicability for short-term mortality prediction in the ICU setting.

**Fig. 4:**
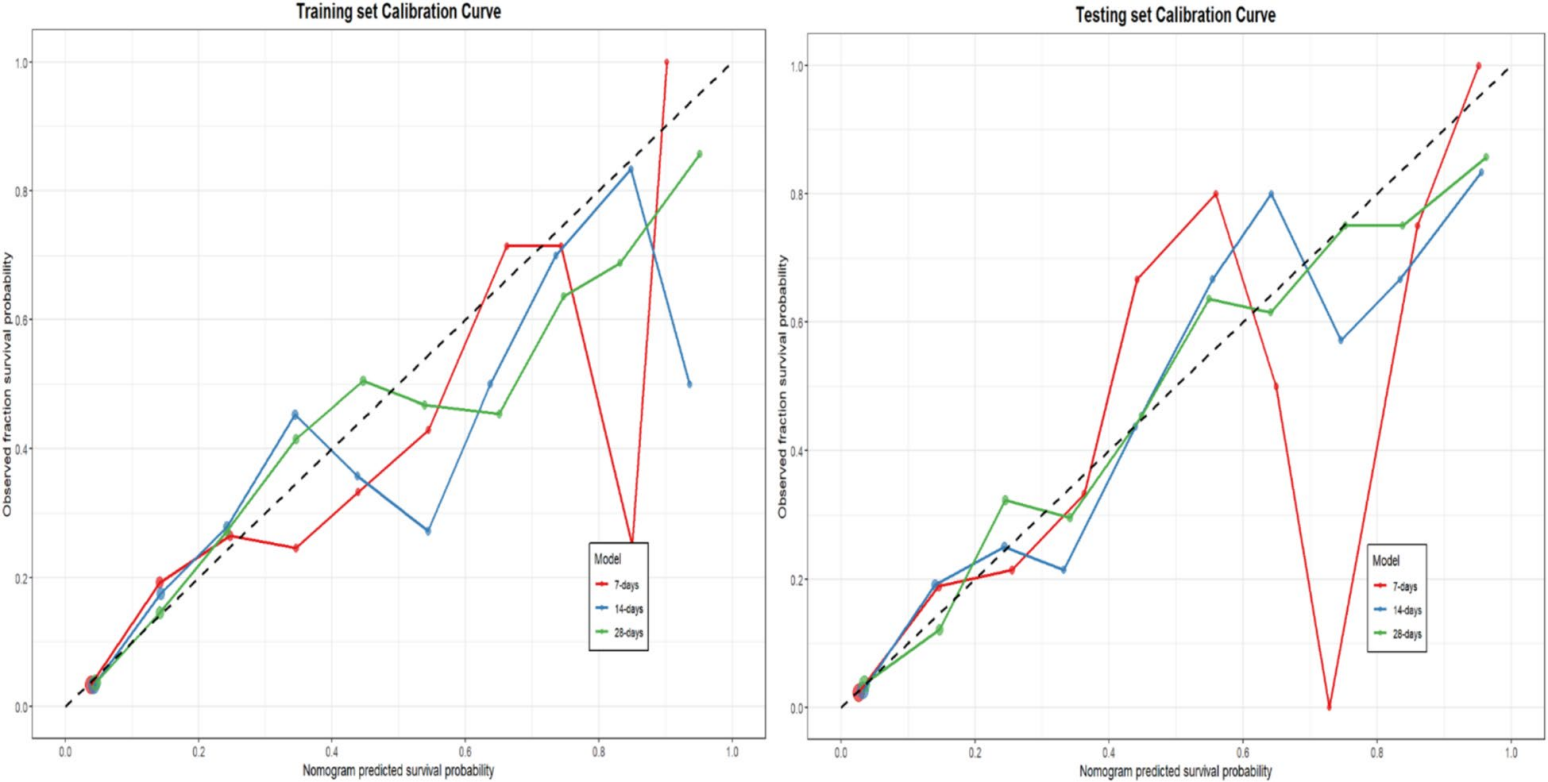
Calibration curves of the nomogram model

The calibration curves demonstrate favorable consistency of the model across both study data sets (Fig. 4). Specifically, in both the training set and testing set, the calibration curves closely align with the ideal reference line (diagonal), visually indicating a high agreement between the model-predicted mortality risk and the actual observed mortality rates. It is worth noting that the model maintains stable calibration performance across the entire range of predicted probabilities, particularly within the intermediate to high-risk ranges. This reinforces our confidence in the reliability of the model's predictions, especially for risk assessment in high-risk patients. Decision curve analysis (DCA) validated the clinical utility of this prediction model at different threshold probabilities.

Following bootstrap internal validation, the predictive model demonstrated excellent performance. It exhibited strong discriminatory power (corrected AUC = 0.8309, 95% CI 0.8241–0.8356) and favorable overall predictive accuracy (Brier score = 0.1126, 95% CI 0.1117–0.1138). Calibration analysis confirmed model reliability: the calibration intercept approached zero (0.0068, 95% CI −0.0078–0.0219), indicating no systematic overestimation or underestimation, while the calibration slope approached unity (0.9622, 95% CI 0.8677–1.0668), demonstrating excellent alignment between predicted probability ranges and actual risk variations. These metrics collectively demonstrate the model's strong clinical applicability, and its predictions can be reliably used to guide clinical decision-making.

The value of each variable corresponds to points on the upper axis, and the total sum of points is projected onto the lower axis to obtain the mortality risk probability.

**Fig. 5:**
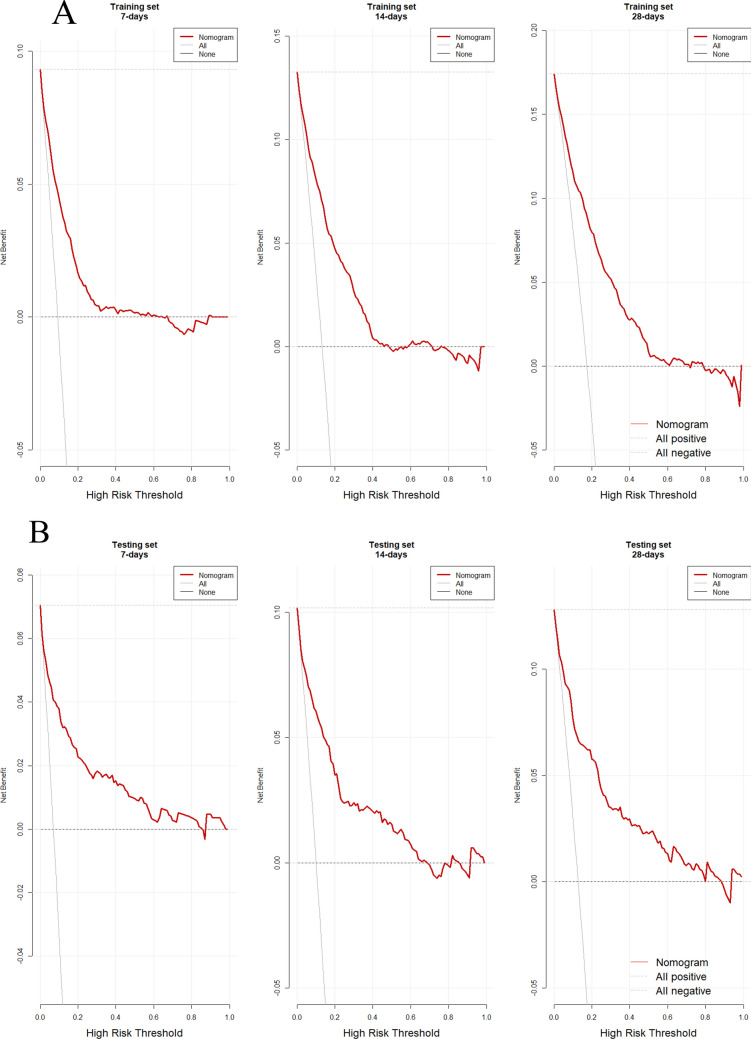
DCA evaluating the clinical utility of the nomogram in the (**A**) training and (**B**) testing sets

The DCA was employed to evaluate the clinical utility of the constructed predictive nomogram. As shown in Fig. 5, we present the decision curves for 7-day, 14-day, and 28-day mortality risk in the training set (Fig. 5A) and the test set (Fig. 5B). The curves compare three clinical strategies: the intervention strategy based on our nomogram, the "treat all patients" strategy, and the "treat no patients" strategy.

The analysis clearly demonstrates that in both the training and test sets, the curve of our model consistently lies above the two reference lines ("treat all" and "treat none") across most clinically meaningful decision threshold ranges. This key finding confirms that using our model to guide clinical decisions yields a higher net benefit compared to the two extreme strategies.

Specifically, the model performs particularly well in short-term prediction (7 days and 14 days), with its curve positioned higher and covering a wider range of threshold probabilities. This indicates its superior clinical value in identifying patients at high near-term risk, effectively assisting clinicians in implementing timely and proactive interventions for these individuals. For 28-day prediction, the model still provides a stable net benefit in the test set, although the range of threshold probabilities, where it demonstrates superiority is somewhat narrower compared to the short-term predictions. This observation aligns with the general pattern that predictive uncertainty increases with longer time horizons.

In summary, the predictive model not only demonstrates discriminative ability from a statistical perspective but, more importantly, is proven to possess significant clinical utility. It shows promise as an effective tool to assist clinicians in risk stratification and precise decision-making for patients with TBI.

### External validation

**Fig. 6:**
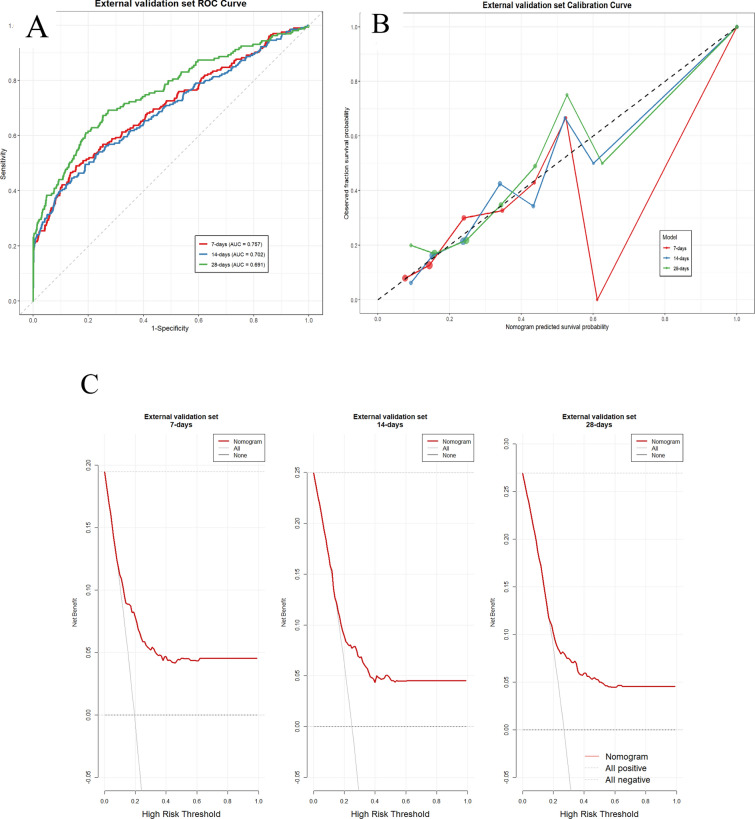
External validation of the nomogram: (**A**) ROC curves, (**B**) calibration curve, and (**C**) decision curve analysis

Conduct external validation to assess the generalization capability of predictive models. During external validation, the model demonstrated robust predictive performance, with the ROC curves (Fig. 6A) showing AUC values of 0.757 for 7-day mortality, 0.702 for 14-day mortality, and 0.691 for 28-day mortality. In the external validation cohort, for the prediction of 28-day mortality, the model proposed in this study demonstrated satisfactory discriminative ability (AUC = 0.691), compared favorably with both the IMPACT core model (AUC = 0.546, unsatisfactory) and the CRASH basic model (AUC = 0.539, unsatisfactory). It is noteworthy that the DeLong test [17] Multiple Replenishments [12] demonstrated statistically significant differences in performance (*p* = 0.006 and *p* = 0.0065, respectively). The predictive performance of all models was evaluated on the same independent external validation set, thereby ensuring a fair comparison. The nomogram demonstrated favorable calibration with slopes of 1.000 and intercepts of 0.000 across all timepoints, along with reliable predictive accuracy evidenced by Brier scores of 0.118, 0.153, and 0.163 for 7-day, 14-day, and 28-day mortality, respectively, collectively confirm the model's satisfactory calibration and clinical utility in external validation. Furthermore, the calibration curves confirmed excellent agreement between predicted and observed outcomes (Fig. 6B), while decision curve analysis demonstrated favorable clinical net benefits across reasonable threshold probabilities (Fig. 6C). These results collectively confirm the model's satisfactory calibration and clinical applicability in external validation.

## Discussion

The study found a 28-day mortality rate of 16.03% for TBI patients, aligning with existing data. Multivariable regression analysis of clinical data from the MIMIC-IV and eICU–CRD databases revealed seven independent predictors significantly linked to mortality in TBI patients. The developed nomogram based on these predictors demonstrates excellent visualization characteristics and enables quantitative prediction of short-term survival outcomes in TBI patients. During validation, the model showed favorable discriminatory performance and clinical translation potential, making it suitable for optimizing risk stratification and supporting individualized clinical decision-making for TBI patients.

The study analyzed 2788 TBI patients from the MIMIC-IV database, identifying seven key prognostic predictors: APS-III score, age, mechanical ventilation use, respiratory rate, prothrombin time, serum sodium levels, and anion gap. Validation results from multiple data sources demonstrated that the model exhibits excellent stability and generalization performance. Internal validation primarily assessed the model's goodness-of-fit in the training cohort, while external validation focused on its performance in new clinical data sets. The results better reflect real clinical settings, greatly improving the model's predictive credibility.

During the external validation phase, the model’s AUC values were consistently lower than those observed during internal validation. This discrepancy in performance can likely be attributed to several differences between the two databases in terms of patient population characteristics and data quality. Notable demographic disparities were observed between the databases, particularly in age, gender, and racial distribution. In addition, systematic differences may exist in disease severity, comorbidity profiles, clinical treatment protocols, and healthcare standards. The MIMIC-IV database consists of single-center data from the Beth Israel Deaconess Medical Center, a national academic medical center, collected between 2008 and 2019. In contrast, the eICU–Collaborative Research Database (eICU–CRD) comprises multicenter data from 208 regional healthcare institutions, collected during 2014–2015. Our model has demonstrated preliminary generalizability across external data sets encompassing diverse healthcare systems. Nevertheless, we recognize that heterogeneity in healthcare practices—such as variations in data collection protocols and equipment—represents a universal challenge that any clinical prediction model must confront when deployed more broadly. Consequently, further validation in more diverse prospective settings is imperative before the model can be implemented in clinical practice. For instance, Beth Israel Deaconess Medical Center often adopts new technologies earlier, while other institutions may continue utilizing conventional technical solutions. This technological disparity ultimately impacts the model's performance across different settings.

Although this study has employed regularization techniques such as LASSO regression during the model development phase to mitigate overfitting, it should be noted that any statistical model still carries the risk of learning specific noise or local patterns present in the development data set. Such patterns often lack generalizability, and when the model is applied to external validation sets with different data distribution characteristics, its predictive performance may consequently attenuate. Notably, the discrepancy between the AUC obtained through bootstrap correction and the AUC observed in the independent external validation set provides crucial quantitative evidence of the degree of this overfitting: it not only reflects the unavoidable optimistic bias during the training process but also reveals the actual boundaries of the model's generalization capability when confronted with out-of-distribution samples.

Multivariable regression analysis identified APS III (Acute Physiology Score III) as an independent predictor of poor prognosis in TBI patients. As a core component of the APACHE II/III system, this scoring system serves as one of the key tools in the critical care field for assessing disease severity and predicting outcomes [18]. Through the integration of 12 physiological parameters (covering basic vital signs, inflammatory markers, and homeostatic parameters), the system enables comprehensive quantitative evaluation of both the risk and severity of multiple organ dysfunction. Compared with APACHE II, APS III features a more streamlined assessment framework. Higher scores typically indicate more significant physiological dysfunction and more severe clinical conditions. In TBI research, elevated APS III scores have been confirmed as significant predictors of adverse outcomes. Multiple empirical studies have also demonstrated that the APACHE scoring system exhibits excellent discriminative efficacy and calibration accuracy in predicting in-hospital mortality.

The anion gap is an essential parameter for diagnosing acid–base imbalances and metabolic acidosis, [19], and it is also a strong prognostic marker in critically ill patients [20]. In the context of TBI, an elevated anion gap likely serves as a composite indicator of the severity of systemic physiological derangement, which can be triggered by hypoperfusion, systemic inflammatory response, or other complications common in severe brain injury [21]. Consequently, its association with poorer outcomes, as validated in this study, underscores the critical impact of extrapolating systemic metabolic distress on the prognosis of TBI patients.

The need for mechanical ventilation itself directly reflects the severity of TBI patients, with most such patients having a GCS score ≤ 8.Prolonged ventilation and associated complications, such as ventilator-associated pneumonia (VAP) and acute respiratory distress syndrome (ARDS), notably elevate mortality risk. Adjustments to ventilation parameters, like PaCO₂ settings, directly affect cerebral blood flow and intracranial pressure, significantly influencing clinical outcomes.

Moreover, parameters including respiratory rate (in both spontaneously breathing and mechanically ventilated patients), prothrombin time (PT), and serum sodium levels serve as robust proxies for the severity of systemic physiological derangement in TBI patients. Abnormalities in these markers are not specific to brain injury but reflect broader complications, such as respiratory failure, coagulopathy, and metabolic dysregulation, which are strongly correlated with increased mortality risk in critical illness. The predictive value of these parameters in our model underscores the significant impact of extrapolating systemic complications on the ultimate prognosis of TBI.

Serum sodium imbalance, whether manifesting as hypernatremia or hyponatremia, is a common metabolic disturbance in critically ill patients and is closely associated with adverse outcomes. In the context of TBI, hypernatremia may result from therapeutic interventions or neuroendocrine dysfunction, while hyponatremia often reflects complex electrolyte dysregulation. Both conditions signify a loss of metabolic homeostasis that can exacerbate cerebral pathophysiology and contribute to increased mortality [22]. The significant difference in sodium levels between outcome groups in this study highlights the prognostic importance of metabolic stability in severe TBI.

Similarly, abnormal PT serves as a marker of systemic coagulation dysfunction [23]. TBI can trigger complex coagulopathies ranging from hypercoagulable states to bleeding tendencies. PT abnormalities reflect this dysregulation and may indicate an elevated risk of hemorrhagic progression or thrombotic complications, both of which can adversely affect patient outcomes [24]. As such, PT represents a valuable indicator of systemic hematological stability with significant implications for TBI prognosis.

The prognostic model developed in this study complements classical models such as CRASH and IMPACT in terms of objectives and application scenarios. While the latter primarily predict long-term neurological outcomes based on baseline admission data, this study focuses on the ICU setting, utilizing dynamic indicators collected after ICU admission to achieve multi-timepoint prediction of short-term mortality risk (7 days, 14 days, and 28 days). This characteristic makes it particularly suitable for guiding real-time clinical decision-making and resource allocation during intensive care.

This study has several limitations. First, while the multi-center origin of the data enhances the generalizability of our findings, the study's primary limitation lies in its reliance on a broad 'TBI' definition without specific inclusion/exclusion criteria for distinct injury subtypes or severities. This approach, while ensuring a large sample size, may introduce heterogeneity that our model cannot fully resolve. Second, as the model was developed using data from U.S. hospitals, external validation in different countries and healthcare systems is essential to confirm its generalizability to other populations. The generalizability of our model requires further validation in cohorts stratified by TBI severity, mechanism, and imaging characteristics.

Third, the absence of key laboratory values and detailed, standardized imaging data—which led to the exclusion of variables with missing rates exceeding 30%—not only potentially introduces selection bias and compromises model robustness but also prevents a direct head-to-head comparison with established prognostic models (e.g., IMPACT and CRASH) that rely on such variables. Fourth, due to its retrospective design, the current model requires prospective validation to confirm its predictive accuracy before it can be applied in clinical practice. Finally, as an observational study, there may be unmeasured or residual confounding factors—such as detailed treatment strategies, specific clinician preferences, or unrecorded comorbidities—that could influence the interpretation of the results.

In addition, we acknowledge limitations regarding model interpretability and potential overfitting. While logistic regression models are generally considered interpretable, the specific contributions and complex interactions of the selected predictors (e.g., APSIII and mechanical ventilation) may not be intuitively clear to clinicians, potentially hindering clinical adoption [25]. Furthermore, despite employing internal validation techniques and LASSO regularization to mitigate overfitting, the risk of the model performing less optimally on entirely independent data sets remains a concern [26]. Future studies should incorporate explainable AI (XAI) techniques, such as SHAP (SHapley Additive exPlanations), to provide more intuitive, patient-level explanations for predictions and strengthen external validation across diverse populations [27–29].

## Conclusion

This study developed and validated a prognostic nomogram for TBI patients, incorporating seven routinely available clinical variables: age, respiratory rate, APS-III score, mechanical ventilation, serum sodium, anion gap, and prothrombin time. In external validation, the model demonstrated statistically significant discriminative ability for short-term mortality compared to existing prognostic models. The nomogram represents a practical visualization of this model. Future research is warranted to evaluate its potential utility in clinical decision-making and whether its implementation could ultimately improve patient outcomes.

## Supplementary Information

Supplementary material 1.

Supplementary material 2.

## Data Availability

The datasets used and/or analysed during the current study are available from the corresponding author on reasonable request.
