# Supplementary material for: Creation and validation of a mortality risk prediction model for ICU patients with traumatic brain injury: a multicenter retrospective cohort study
Source: Eur J Med Res. 2025 Dec 9;30:1242. doi: 10.1186/s40001-025-03656-3 (PMC12715908; doi:10.1186/s40001-025-03656-3)

**Table S1** **Training Set Baseline Features**

| **Variable** | **Survival（n=1612）** | **Non- Survival(n=340)** | **P-Value** |
| --- | --- | --- | --- |
| **Sex** | | | |
| Male | 577 (35.8%) | 154 (45.3%) | 0.001 |
| Female | 1035 (64.2%) | 186 (54.7%) |  |
| **Race** | | | |
| Caucasians | 991 (61.5%) | 185 (54.4%) | 0.018 |
| Other | 621 (38.5%) | 155 (45.6%) |  |
| **Age (years)** | 59.67 ±21.16 | 70.62 ±19.36 | ＜0.001 |
| **PT(s)** | 13.29 ±3.65 | 15.51±6.61 | ＜0.001 |
| **PTT(s)** | 28.68 ±7.43 | 31.54 ±10.63 | ＜0.001 |
| **Glucose (mmol/L)** | 7.38 ±2.69 | 8.64±3.53 | ＜0.001 |
| **Na^+^ (mmol/L)** | 139.01 ±4.18 | 140.05 ±5.32 | 0.001 |
| **K^+^ (mmol/L)** | 4.08±0.52 | 4.19±0.64 | 0.002 |
| **HCO₃⁻ (mmol/L)** | 20.41±6.17 | 23.48±8.75 | ＜0.001 |
| **Cl^-^ (mmol/L)** | 104.01±5.17 | 105.09±6.43 | 0.004 |
| **RBC (x10⁶/µL)** | 3.66±0.72 | 3.45±0.7 | ＜0.001 |
| **HR (minute)** | 81.99±15.33 | 85.54±16.43 | ＜0.001 |
| **SBP (mmHg)** | 124.55±13.29 | 123.64±14.58 | 0.291 |
| **DBP (mmHg)** | 66.27±10.44 | 62.62±10.53 | ＜0.001 |
| **RR (minute)** | 18.15±2.95 | 19.63±3.71 | ＜0.001 |
| **T（℃）** | 37.05±0.45 | 37±0.67 | 0.153 |
| **Spo2（%）** | 97.46±1.76 | 97.88±2.1 | 0.001 |
| **APSIII score** | 36.01±14.83 | 51.1±22.34 | ＜0.001 |
| **OASIS score** | 30.54±7.26 | 36.55±7.25 | ＜0.001 |
| **GCS score** | 13.16±2.47 | 12.29±3.87 | ＜0.001 |
| **CCI score** | 3.25±2.79 | 4.88±2.98 | ＜0.001 |
| **Platelets (x10³/µL)** | 206.48±76.53 | 190.61±86.25 | 0.002 |
| **WBC (x10³/µL)** | 11.37±6 | 13.09±7.14 | ＜0.001 |
| **AG (mEq/L)** | 14.24±3.11 | 15.42±3.44 | ＜0.001 |
| **Creatinine (mg/dL)** | 1.03±0.88 | 1.27±0.97 | ＜0.001 |
| **BUN (mg/dL)** | 17.4±11.96 | 24.68±17.43 | ＜0.001 |
| **Mechanical** | | | |
| NO | 955 (59.2%) | 113 (33.2%) | ＜0.001 |
| YES | 657 (40.8%) | 227 (66.8%) |  |
| **CHF** | | | |
| NO | 1419 (88%) | 269 (79.1%) | ＜0.001 |
| YES | 193 (12%) | 71 (20.9%) |  |
| **CVD** | | | |
| NO | 1448 (89.8%) | 291 (85.6%) | 0.029 |
| YES | 164 (10.2%) | 49 (14.4%) |  |
| **Chronic lung disease** | | | |
| NO | 1409 (87.4%) | 294 (86.5%) | 0.703 |
| YES | 203 (12.6%) | 46 (13.5%) |  |
| **Malignant** | | | |
| NO | 1558 (96.7%) | 318 (93.5%) | 0.011 |
| YES | 54 (3.3%) | 22 (6.5%) |  |
| **Liver** | | | |
| NO | 1520 (94.3%) | 309 (90.9%) | 0.026 |
| YES | 92 (5.7%) | 31 (9.1%) |  |
| **MST** | | | |
| NO | 1598 (99.1%) | 331 (97.4%) | 0.011 |
| YES | 14 (0.9%) | 9 (2.6%) |  |
| **AKI** | | | |
| NO | 542 (33.6%) | 50 (14.7%) | ＜0.001 |
| YES | 1070 (66.4%) | 290 (85.3%) |  |

**HR: Heart rate; RR: Respiratory rate ;DBP: Diastolic blood pressure; SBP: Systolic blood pressure; SpO₂: Blood oxygen saturation; CCI: Charlson comorbidity index; WBC: White blood cell count; RBC: Red blood cell count; BUN: Blood urea nitrogen; CHF: Congestive Heart Failure; WBC: White Blood Cell (count); PT: Prothrombin Time; PTT: Partial Thromboplastin Time; MST: metastatic solid tumor; AKI, acute kidney injury; CVD：Cerebrovascular disease;** **AG: Anion-gap**

**Table S2 Differences between training and test groups Baseline characteristics**

| **Variable** | **Training set(n=1952)** | **Testing set(n=836)** | **P-Value** |
| --- | --- | --- | --- |
| **Sex** | | | |
| Male | 731 (37.4%) | 282 (33.7%) | 0.068 |
| Female | 1221 (62.6%) | 554 (66.3%) |  |
| **Race** | | | |
| Caucasians | 1176 (60.2%) | 530 (63.4%) | 0.128 |
| Other | 776 (39.8% | 306 (36.6%) |  |
| **Age (years)** | 61.58±21.27 | 60.49±21.44 | 0.22 |
| **PT(s)** | 13.68±4.4 | 13.72±4.34 | 0.817 |
| **PTT(s)** | 29.18±8.15 | 29.38±8.08 | 0.563 |
| **Glucose (mmol/L)** | 7.6 ±2.89 | 7.78±3.67 | 0.227 |
| **Na^+^ (mmol/L)** | 139.19±4.41 | 139.36±4.24 | 0.339 |
| **K^+^ (mmol/L)** | 4.1±0.55 | 4.14±0.55 | 0.067 |
| **HCO₃⁻ (mmol/L)** | 20.94±6.79 | 20.81±6.75 | 0.625 |
| **Cl^-^ (mmol/L)** | 104.2±5.42 | 104.32±5.37 | 0.608 |
| **RBC (x10⁶/µL)** | 3.63±0.72 | 3.7±0.72 | 0.021 |
| **HR (minute)** | 82.61±15.58 | 82.47±15.74 | 0.825 |
| **SBP (mmHg)** | 124.39±13.52 | 124.59±14.08 | 0.735 |
| **DBP (mmHg)** | 65.64±10.55 | 66.12±10.77 | 0.274 |
| **RR (minute)** | 18.41±3.14 | 18.49±3.23 | 0.538 |
| **T（℃）** | 37.04±0.5 | 37.04±0.5 | 0.716 |
| **Spo2（%）** | 97.53±1.83 | 97.45±1.71 | 0.259 |
| **APSIII score** | 38.64±17.35 | 37.59±16.43 | 0.128 |
| **OASIS score** | 31.58±7.61 | 31.13±7.3 | 0.135 |
| **GCS score** | 13.01±2.78 | 13.12±2.59 | 0.305 |
| **CCI score** | 3.54±2.89 | 3.28±2.73 | 0.023 |
| **Platelets (x10³/µL)** | 203.72±78.52 | 204.28±88.97 | 0.873 |
| **WBC (x10³/µL)** | 11.67±6.25 | 11.62±5.48 | 0.824 |
| **Anion-gap (mEq/L)** | 14.45±3.2 | 14.56±3.1 | 0.376 |
| **Creatinine (mg/dL)** | 1.07±0.9 | 1.08±0.94 | 0.797 |
| **BUN (mg/dL)** | 18.67±13.36 | 18.45±13.34 | 0.685 |
| **Mechanical** |  |  |  |
| NO | 1068 (54.7%) | 473 (56.6%) | 0.386 |
| YES | 884 (45.3%) | 363 (43.4%) |  |
| **CHF** |  |  |  |
| NO | 1688 (86.5%) | 756 (90.4%) | 0.004 |
| YES | 264 (13.5%) | 80 (9.6%) |  |
| **CVD** |  |  |  |
| NO | 1739 (89.1%) | 757 (90.6%) | 0.277 |
| YES | 213 (10.9%) | 79 (9.4%) |  |
| **Chronic lung disease** |  |  |  |
| NO | 1703 (87.2%) | 742 (88.8%) | 0.293 |
| YES | 249 (12.8%) | 94 (11.2%) |  |
| **Malignant** |  |  |  |
| NO | 1876 (96.1%) | 807 (96.5%) | 0.666 |
| YES | 76 (3.9%) | 29 (3.5%) |  |
| **Liver** |  |  |  |
| NO | 1829 (93.7%) | 785 (93.9%) | 0.908 |
| YES | 123 (6.3%) | 51 (6.1%) |  |
| **MST** |  |  |  |
| NO | 1929 (98.8%) | 829 (99.2%) | 0.549 |
| YES | 23 (1.2%) | 7 (0.8%) |  |
| **AKI** |  |  |  |
| NO | 592 (30.3%) | 280 (33.5%) | 0.108 |
| YES | 1360 (69.7%) | 556 (66.5%) |  |

**HR: Heart rate; RR: Respiratory rate ;DBP: Diastolic blood pressure; SBP: Systolic blood pressure; SpO₂: Blood oxygen saturation; CCI: Charlson comorbidity index; WBC: White blood cell count; RBC: Red blood cell count; BUN: Blood urea nitrogen; CHF: Congestive Heart Failure; WBC: White Blood Cell (count); PT: Prothrombin Time; PTT: Partial Thromboplastin Time; MST: metastatic solid tumor; AKI, acute kidney injury; CVD：Cerebrovascular disease;** **AG: Anion-gap**


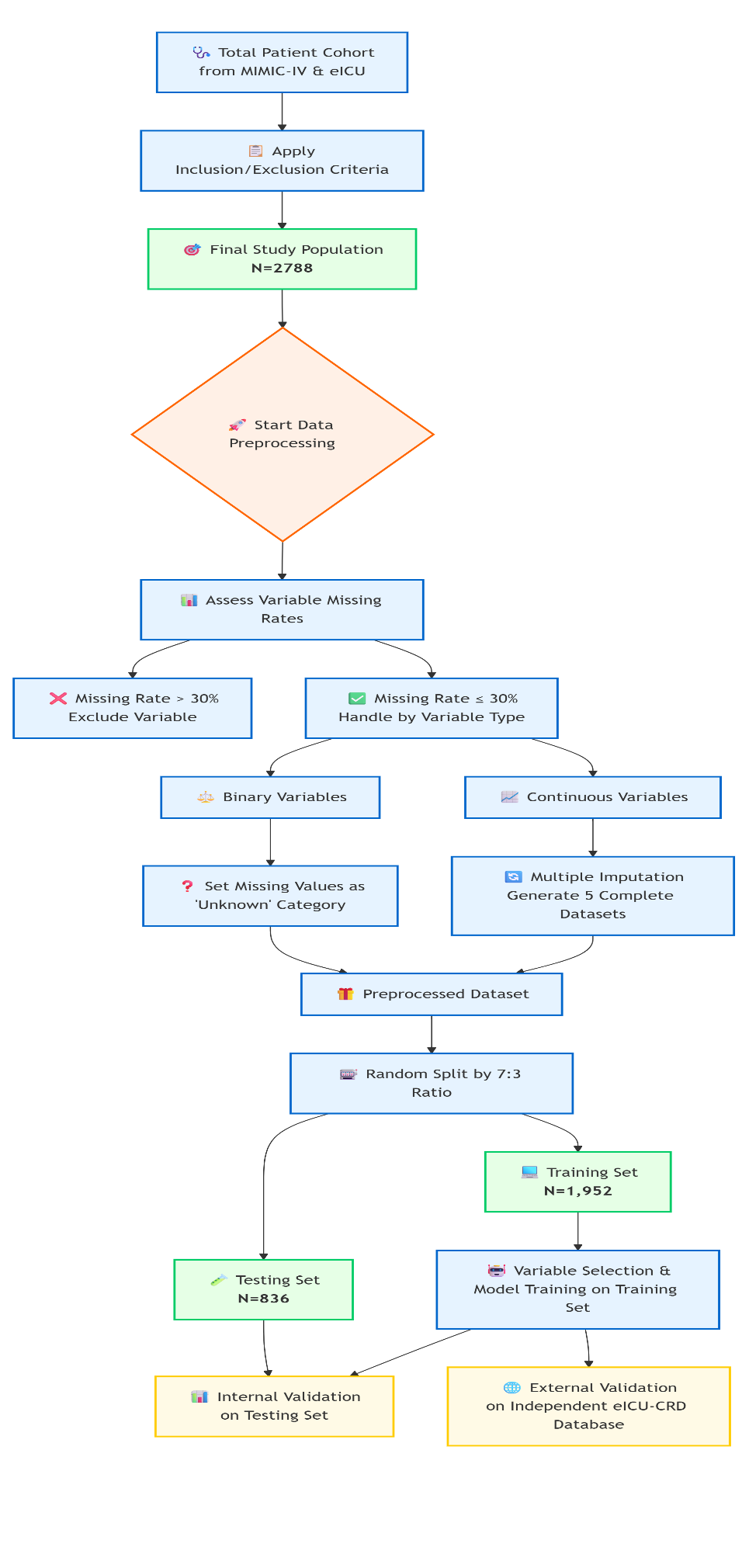
**Figure S1. Workflow of Data Cleaning and Modeling Process.**

**Figure S2. LASSO Cross-Validation Plot**


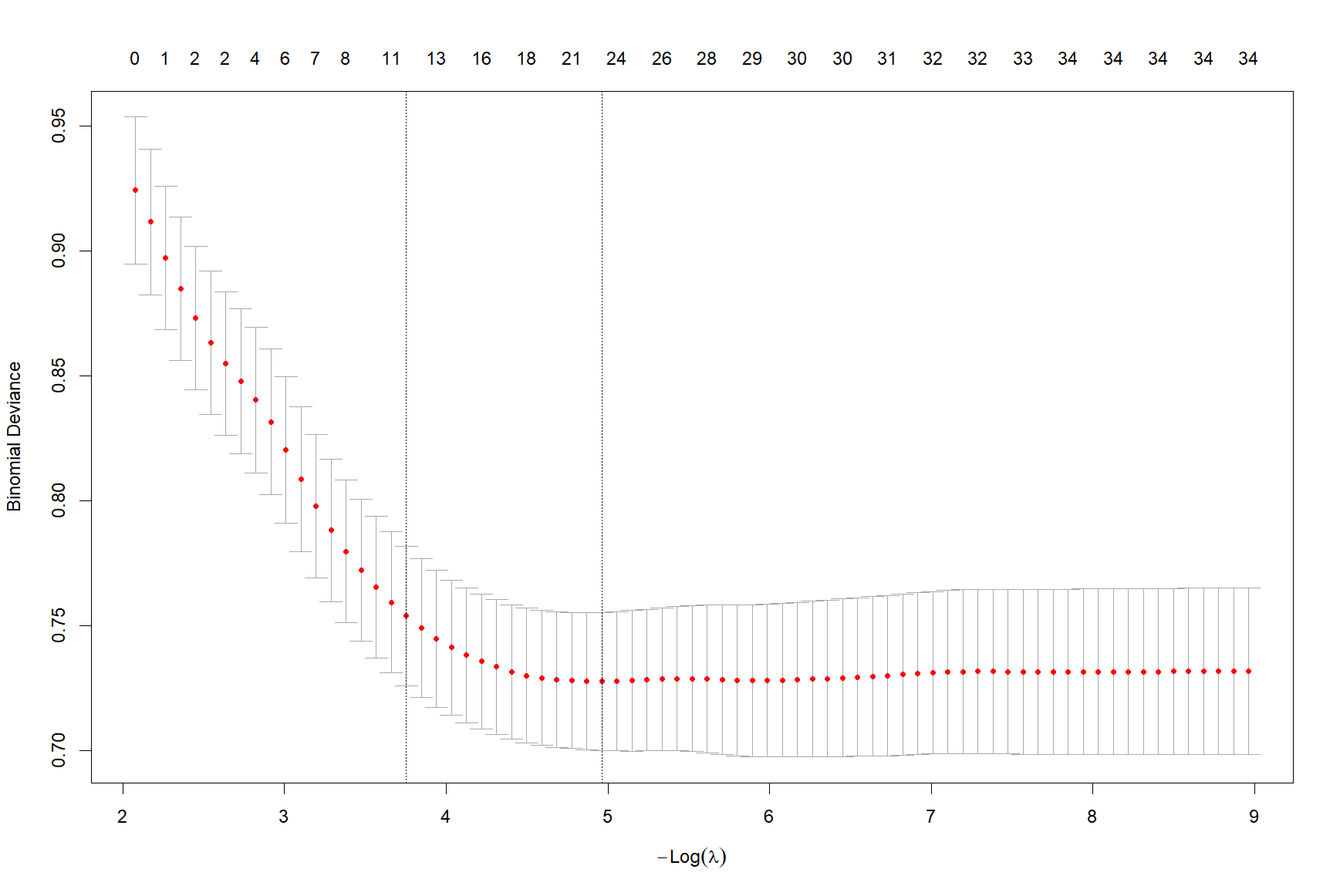


**Figure S3. Variable Importance Plot for LASSO-Selected Features.**


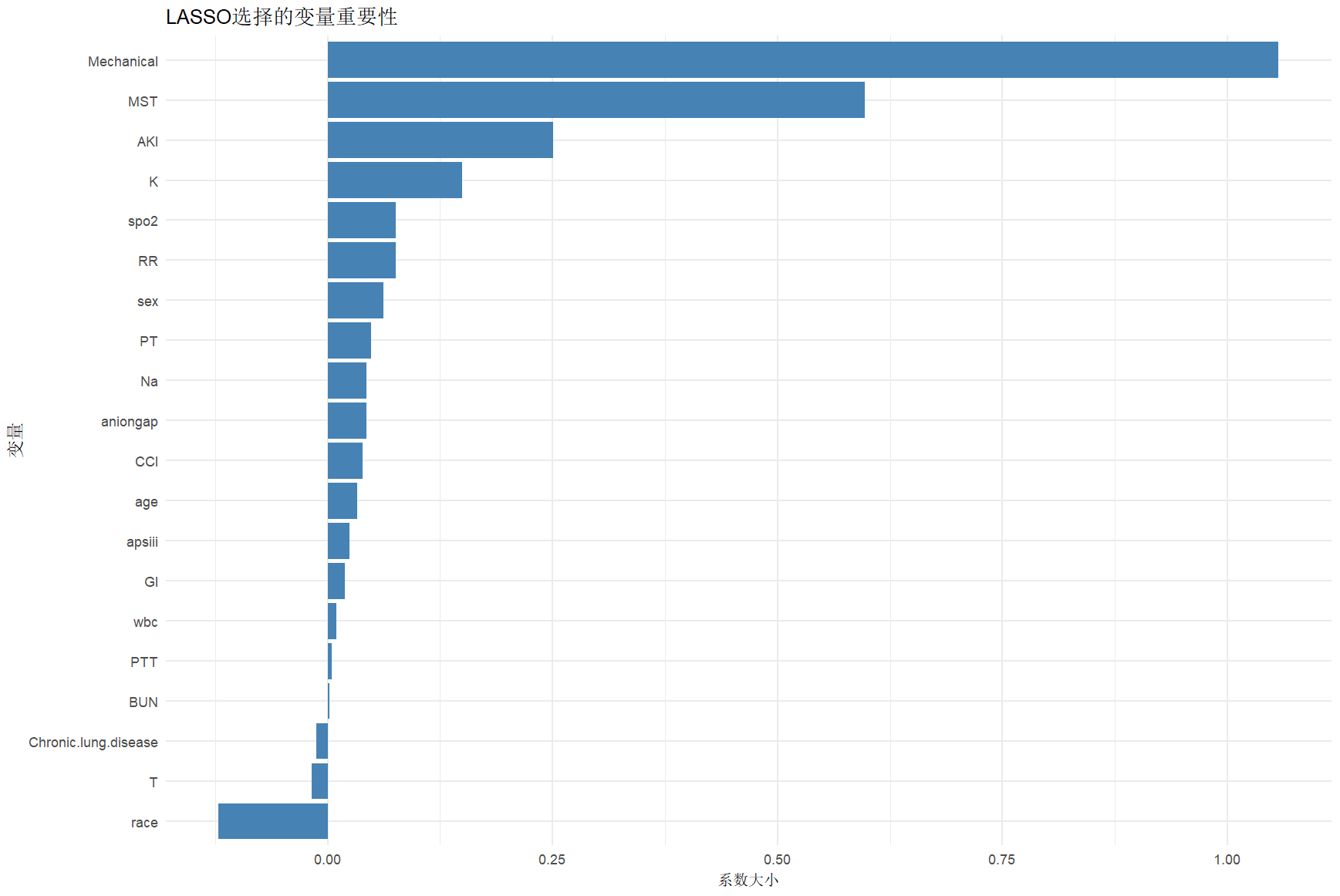

Supplement: Supplementary file 1 — Supplementary material 1. [file 40001_2025_3656_MOESM1_ESM.docx]
