# Supplementary material for: Creation and validation of a mortality risk prediction model for ICU patients with traumatic brain injury: a multicenter retrospective cohort study
Source: Eur J Med Res. 2025 Dec 9;30:1242. doi: 10.1186/s40001-025-03656-3 (PMC12715908; doi:10.1186/s40001-025-03656-3)
Supplement: Supplementary file 2 — Supplementary material 2. [file 40001_2025_3656_MOESM2_ESM.docx]

**Complete Model Coefficients:**

| **Variable** | **7-day Coefficient** | **14-day Coefficient** | **28-day Coefficient** |
| --- | --- | --- | --- |
| **Intercept** | -22.5443 | -20.9162 | -18.2122 |
| **APSIII** | 0.0244 | 0.0277 | 0.0298 |
| **Age** | 0.0284 | 0.0369 | 0.0430 |
| **Mechanical** | 1.6191 | 1.6687 | 1.4054 |
| **RR** | 0.0627 | 0.0710 | 0.0911 |
| **PT** | 0.0335 | 0.0367 | 0.0704 |
| **Na^+^** | 0.1015 | 0.0840 | 0.0578 |
| **AG** | 0.0358 | 0.0585 | 0.0699 |

**Complete Prediction Formulas:**

**7-day Mortality Prediction:**
logit(P) = -22.5443 + 0.0244×APSIII + 0.0284×Age + 1.6191×Mechanical + 0.0627×RR + 0.0335×PT + 0.1015×Na + 0.0358×AG
P = 1 / (1 + exp(-logit(P)))

**14-day Mortality Prediction:**
logit(P) = -20.9162 + 0.0277×APSIII + 0.0369×Age + 1.6687×Mechanical + 0.0710×RR + 0.0367×PT + 0.0840×Na + 0.0585×AG
P = 1 / (1 + exp(-logit(P)))

**28-day Mortality Prediction:**
logit(P) = -18.2122 + 0.0298×APSIII + 0.0430×Age + 1.4054×Mechanical + 0.0911×RR + 0.0704×PT + 0.0578×Na + 0.0699×AG
P = 1 / (1 + exp(-logit(P)))
